# ApoA-I mimetics favorably impact cyclooxygenase 2 and bioactive lipids that may contribute to cardiometabolic syndrome in chronic treated HIV

**DOI:** 10.1016/j.metabol.2021.154888

**Published:** 2021-09-09

**Authors:** M. Daskou, M. Sharma, W. Mu, R. Heymans, E. Ritou, V. Rezek, P. Hamid, A. Kossyvakis, S. Sen Roy, V. Grijalva, A. Chattopadhyay, J. Papesh, D. Meriwether, S.G. Kitchen, A.M. Fogelman, S.T. Reddy, T. Kelesidis

**Affiliations:** a Department of Medicine, Division of Infectious Diseases, David Geffen School of Medicine, University of California Los Angeles, Los Angeles, CA, USA; b Department of Medicine, Division of Hematology and Oncology, David Geffen School of Medicine, University of California Los Angeles, Los Angeles, CA, USA; c Department of Medicine, Division of Cardiology, David Geffen School of Medicine, University of California Los Angeles, Los Angeles, CA, USA; d Department of Molecular and Medical Pharmacology, University of California Los Angeles, Los Angeles, CA, USA; e Molecular Toxicology Interdepartmental Degree Program, University of California Los Angeles, Los Angeles, CA, USA

**Keywords:** ApoA-I mimetic peptides, Eicosanoids, Bioactive lipids, Inflammation, Cardiometabolic syndrome, Chronic treated HIV

## Abstract

**Objective:**

We investigated whether apolipoprotein A-I (apoA-I) mimetic peptides 4F and 6F can be a novel therapeutic strategy to reduce blood and gut bioactive lipids, proinflammatory effects of endotoxin (LPS) and aberrant activation of cyclooxygenase 2 (COX-2) as instigators of increased risk for cardiometabolic disease in chronic treated HIV.

**Methods:**

We used two humanized murine models of chronic treated HIV infection (*n* = 109 mice) and gut explants from HIV infected (n = 10) persons to determine whether Tg6F and 4F attenuate *in vivo* and *ex vivo* increased blood and gut bioactive lipids (measured by mass spectrometry) and intestinal protein levels of COX-2 (measured by immunoassays) in chronic treated HIV.

**Results:**

In these models of HIV, when compared to HIV-1 infected mice on antiretroviral therapy (ART) alone, oral Tg6F in combination with ART attenuated increases in plasma and gut bioactive lipids (and particularly COX lipids) and intestinal COX-2. 4F and Tg6F also reduced *ex vivo* production of COX-2 protein and associated secretion of bioactive lipids in gut explants from HIV-1 infected persons treated with LPS.

**Conclusion:**

ApoA-I mimetics favorably impact the proinflammatory effects of LPS, COX-2 and production of bioactive lipids that collectively drive gut and systemic inflammation in chronic treated HIV. Given prior experimental evidence that the proinflammatory effects of LPS, COX-2 and gut dysfunction contribute to cardiometabolic syndrome in chronic HIV, apoA-I mimetic peptides may be a novel therapy to treat cardiometabolic syndrome in chronic HIV.

## 1. Introduction

Chronic treated HIV is a state of gut barrier dysfunction in which HIV, antiretroviral therapy (ART), gut cells, microbial products such as lipopolysaccharides (LPS) and bioactive lipids interact to drive increased systemic and intestinal inflammation, immune dysfunction and metabolic syndrome [1]. However, the exact mechanisms of increased risk for cardiometabolic disease in chronic treated HIV are unclear. Pending results from clinical trials, it is unclear whether statins can prevent metabolic syndrome and morbidity in chronic treated HIV [2]. Thus, there is a tremendous unmet need for novel therapies to reduce metabolic abnormalities and morbidity in chronic treated HIV. Identifying the mechanisms that drive intestinal inflammation, LPS, bioactive lipids and gut barrier dysfunction may lead to novel therapies for chronic treated HIV-related metabolic comorbidities.

Altered lipids are associated with impaired immune responses, comorbidities and aging in people with chronic treated HIV [3,4]. Although dyslipidemia may reflect altered metabolism in HIV [5], certain bioactive lipids like fatty acids may directly contribute to insulin resistance and the pathogenesis of metabolic syndrome [6,7]. However, little is known about the impact of HIV-1 *versus* ART on bioactive lipids such as prostanoids, a family of lipid mediators generated by the action of cyclooxygenase (COX) on unsaturated fatty acids [8]. COX-2 is the more important source of prostanoids in inflammatory conditions and has a key role in pathogenesis of several diseases including cardiovascular disease [8], obesity and the metabolic syndrome [9]. Although COX-2 is present in all the cells involved in HIV end organ disease including immune, endothelial and epithelial cells [8], the role of COX-2 and associated prostanoids in inflammation and gut barrier dysfunction as instigators of metabolic syndrome specifically in HIV remain unclear. Gut and other tissues are not readily available from HIV infected persons for immediate analysis of COX-2 protein levels and unstable bioactive lipids that need specialized laboratory procedures. Herein, to bypass these limitations, we used a translational robust approach with two independent humanized mouse models of chronic ART treated HIV, to mechanistically characterize the direct impact of HIV-1 *per se* and ART on formation of bioactive lipids in blood and intestine of mice with chronic treated HIV.

Given that mechanistic animal models demonstrated that bioactive lipids and COX-2 are involved in the pathogenesis of inflammation and can be targeted therapeutically [10,11], we hypothesized that apolipoprotein A-I (apoA-I) mimetic peptides, which bind bioactive lipids with higher affinity than full length apoA-I, target COX-2 [11] and have shown activity in animal models of chronic inflammatory diseases [12], may be a novel therapeutic strategy that can reduce increased bioactive lipids and COX-2 in chronic HIV. One of these peptides named 6F that is expressed as a transgene in tomatoes, when concentrated (Tg6F) and administered orally, works mainly in the gut to inhibit gut inflammation and progression of inflammatory diseases in mice [11,13,14]. In this report, by using a previously described validated translational approach with independent humanized mouse models of chronic treated HIV and human gut explants from HIV infected persons that showed that that the apoA-I mimetic peptides 4F and 6F attenuate biomarkers of immune activation [15], we also demonstrate that apoA-I mimetics attenuate increased blood and gut bioactive lipids and intestinal COX-2 in chronic treated HIV. Our data provide proof of concept that apoA-I mimetic peptides may be a novel therapeutic strategy to target intestinal COX-2 and bioactive lipids as instigators of inflammation, gut barrier dysfunction and cardiometabolic syndrome in chronic treated HIV.

## 2. Methods

### 2.1. Materials

The antivirals Emtricitabine (FTC), Tenofovir Disoproxil Fumarate (TDF), Raltegravir (RAL), abacavir (ABC), dolutegravir (DOL), lamivudine (3TC), tenofovir alafenamide (TAF) were a gift from Gilead Sciences or were obtained from pharmacy. HIV p89.6 plasmid DNA was obtained from the NIH AIDS reagent Program. Murine chow diet with antibiotic was purchased from Envigo Teklad Diets. All other materials were purchased from commercially available sources and have been previously described in detail [15]. All lipids were purchased from Cayman Chemicals (Ann Arbor, MI, USA) and are described in detail in the Supplemental material.

### 2.2. Mice

All animal protocols were carried out in accordance with all federal, state, and local approved guidelines. Graft *versus* host disease (GVHD)-resistant C57BL/6 recombination activating gene 2 (Rag2)γcCD47 triple knockout (TKO) BLT mice or NOD.Cg-*Prkdc*^*scid*^*Il2rg*^*tm1Wjl*^/SzJ (NSG) Bone Marrow Liver Thymic mice (BLT) mice were all generated and maintained as described [15]. A total of 5 (2 TKO and 3 NSG) independent cohorts of mice were pooled for comparing mice that were uninfected (HIV^−^), infected and viremic (HIV^+^), infected and on potent ART (HIV^+^ART^+^) and infected on potent ART and Tg6F (HIV^+^ART^+^Tg6F^+^) (*n* = 8–20 mice per group). Given the variable engraftment of human immune cells and to better compare results among mice, we determined changes in measures among mice groups over 16 weeks of potent ART and we compared them to the uninfected group within each cohort (identical human donor). All data from independent cohorts were pooled together. In the TKO BLT mice, Tg6F was given the day after the viral load was found to be suppressed while in the NSG BLT mice it was initiated 2 weeks before confirmation of viremia suppression. The current study used murine biospecimens (blood and gut tissues) from a previously published study [15] to generate novel experimental data described in this study.

### 2.3. HIV infection

Between 12 and 18 weeks after humanization, mice were challenged intraperitoneally with 500 ng p24 of HIV-1 dual-tropic 89.6 virus and plasma HIV-1 viral load as described previously [15].

### 2.4. ART treatment

ART was a combination of either ABC/DOL/3TC (Triumeq)(TKO) or TDF/FTC/RAL as previously described [15].

### 2.5. Mouse blood and murine and human gut tissue processing

Murine blood and gut tissues were processed as described previously [15] or as described in the Supplemental material.

### 2.6. Determination of bioactive lipids

Bioactive lipids were determined as previously descried [11] using multiple reaction monitoring liquid chromatography with tandem mass spectrometry (LC-MS-MS) in negative ion mode coupled together with liquid chromatography that covers 39 distinct bioactive lipids, degradation products, and pathway markers. Further details are described in the Supplemental material.

### 2.7. Study participants

HIV-seronegative healthy (*n* = 10) and HIV seropositive (n = 10) 50–60 year old men on potent ART with suppressed viremia, CD4^+^ T cells >500 cells/mm^3^, with no known cardiometabolic risk factors or clinical disease other than HIV were recruited as previously described [15]. All individuals enrolled in the study provided written informed consent and the study was approved by the local Institutional Review Board. The current study used human biospecimens (gut tissues) from a previously published study [15] to generate novel experimental data described in this study.

### 2.8. Gut explants

Gut biopsies were obtained endoscopically from the colon and gut explants were processed as previously described [15]. Triplicate biopsies were treated with media (control) and/or with LPS at concentration 100 μg/ml [16] and/or with 4F, 6F for 72 h as previously described [11,16,17].

### 2.9. ApoA-I mimetics

Mice were administered a concentrate of control transgenic tomatoes (EV) or a concentrate of transgenic tomatoes expressing the 6F peptide (Tg6F). Both concentrates were added to the diet at 0.06% by weight and fed to the mice for 12 weeks as previously described [13]. 4F and 6F peptides were synthesized as described previously [11]. Triplicate biopsies were treated with 4F, 6F, or sham peptide at a concentration 100 μg/ml for 72 h as previously described [11].

### 2.10. Immunoassays

Gut tissue human and murine COX-2 protein levels and 6-keto-PGF1α (6kPGF1α) were determined using ELISA assay kits according to the manufacturer’s instructions (MyBiosource or Cayman Chemicals).

### 2.11. Flow cytometry

Flow cytometry was performed in gut tissue single cell suspensions, as described in the Supplemental material and as previously reported [15].

### 2.12. Statistics

*P* values less than 0.05 by Kruskal-Wallis or Mann-Whitney were considered significant. For all correlations, Spearman’s correlation coefficient was calculated. All analyses were performed with GraphPad Prism, version 7.0. Further details are described in the Supplemental material.

## 3. Results

### 3.1. HIV-1 increases plasma and gut levels of bioactive lipids in vivo

Humanized BLT mice infected with HIV are an established model of HIV-1 immunopathogenesis *in vivo* [18]. We generated two independent (TKO and NSG) BLT models of humanized mice with identical functional human immune cells within each cohort that were mock-infected or infected with HIV-1 (HIV^+^) and treated with independent contemporary ART regimens that similarly suppressed plasma viremia in both BLT models [15]. We have shown no differences in plasma levels of lipids among all groups in both BLT models [15]. We hypothesized that plasma and gut bioactive lipids of the proinflammatory Arachidonic acid (AA) pathways and other major pathways of eicosanoids would be increased in chronic treated HIV and could be targeted therapeutically with apoA-I mimetic peptides.

**Fig. 1.**
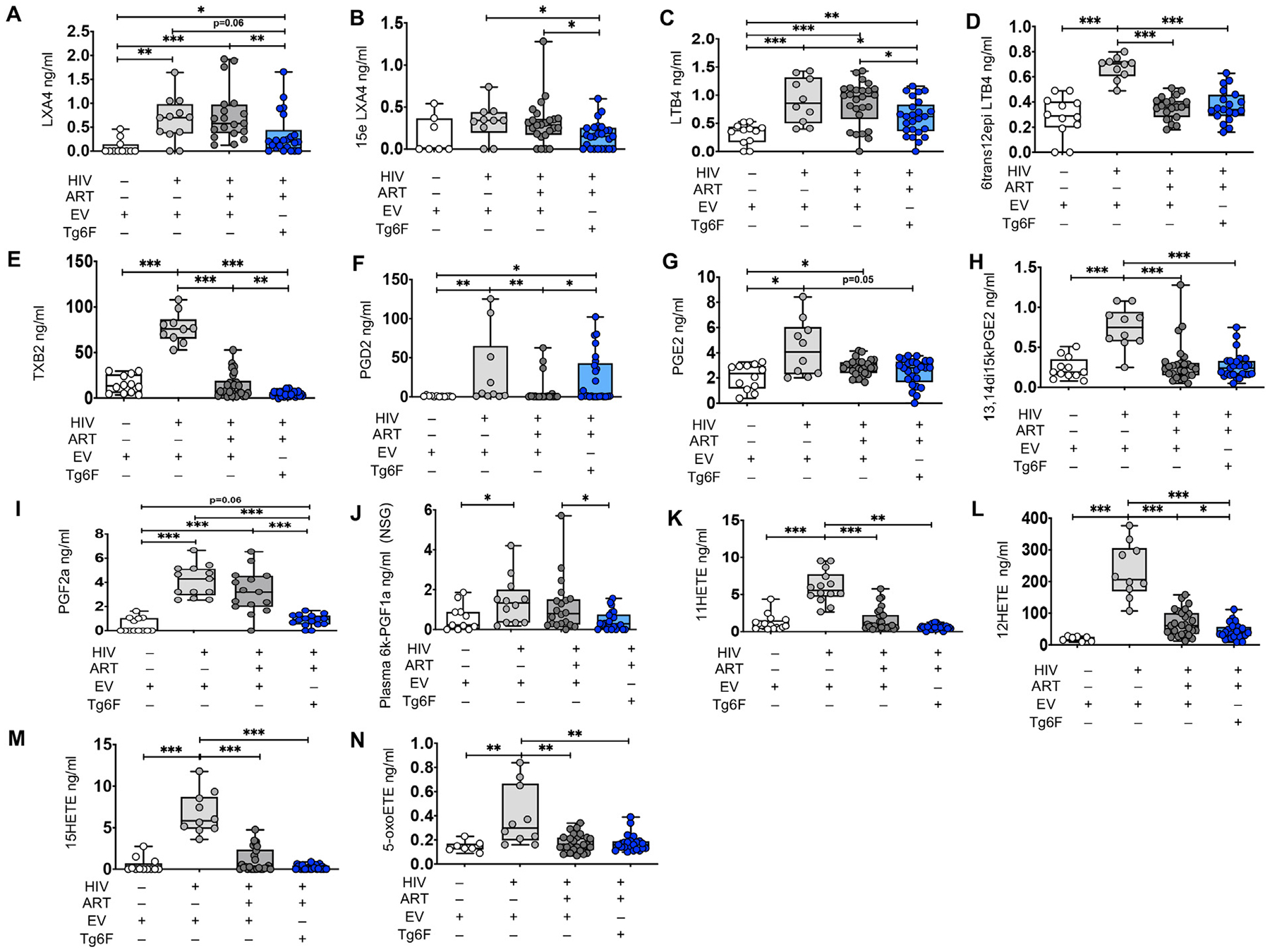
Tg6F attenuates alterations in plasma bioactive lipids of the Arachidonic acid pathway driven by HIV and/or ART in a NSG humanized mouse model of chronic treated HIV infection. NSG (*n* = 64) BLT mice were constructed, infected with HIV and treated with ART, control transgenic tomato concentrate (EV) or Tg6F as described in Methods. Plasma bioactive lipids of the Arachidonic acid (AA) pathways were determined by multiple reaction monitoring (MRM) Liquid Chromatography with tandem mass spectrometry (LC-MS-MS) as described in Methods (A–M). Data represent box and whiskers with minimum, median and maximum values of lipoxin A4 (LXA4) (A), 15-epi-lipoxin A4 (15epi-LXA4) (B), leukotriene B4 (LTB4) (C), 6-trans-12-epi leukotriene B4 (6trans-12epi-LTB4) (D), thromboxane B2 (TXB2) (E), prostaglandin D2 (PGD2) (F), prostaglandin E2 (PGE2) (G), 13,14di-15-keto-prostaglandin E2 (13,14di15kPGE2) (H), prostaglandin F2α (PGF2α) (I), plasma 6-keto-prostaglandin F1α (6kPGF1α) (J), 11-hydroxyeicosatetraenoic acid (11-HETE) (K), 12-hydroxyeicosatetraenoic acid (12-HETE) (L), 15-hydroxyeicosatetraenoic acid (15-HETE) (M), 5-oxoeicosatetraenoic acid (5-oxoETE) (N). The Mann-Whitney test was used to compare 2 groups (*n* = 8–22 mice per group) (*p < 0.05, **p < 0.01, ***p < 0.001).

**Fig. 2.**
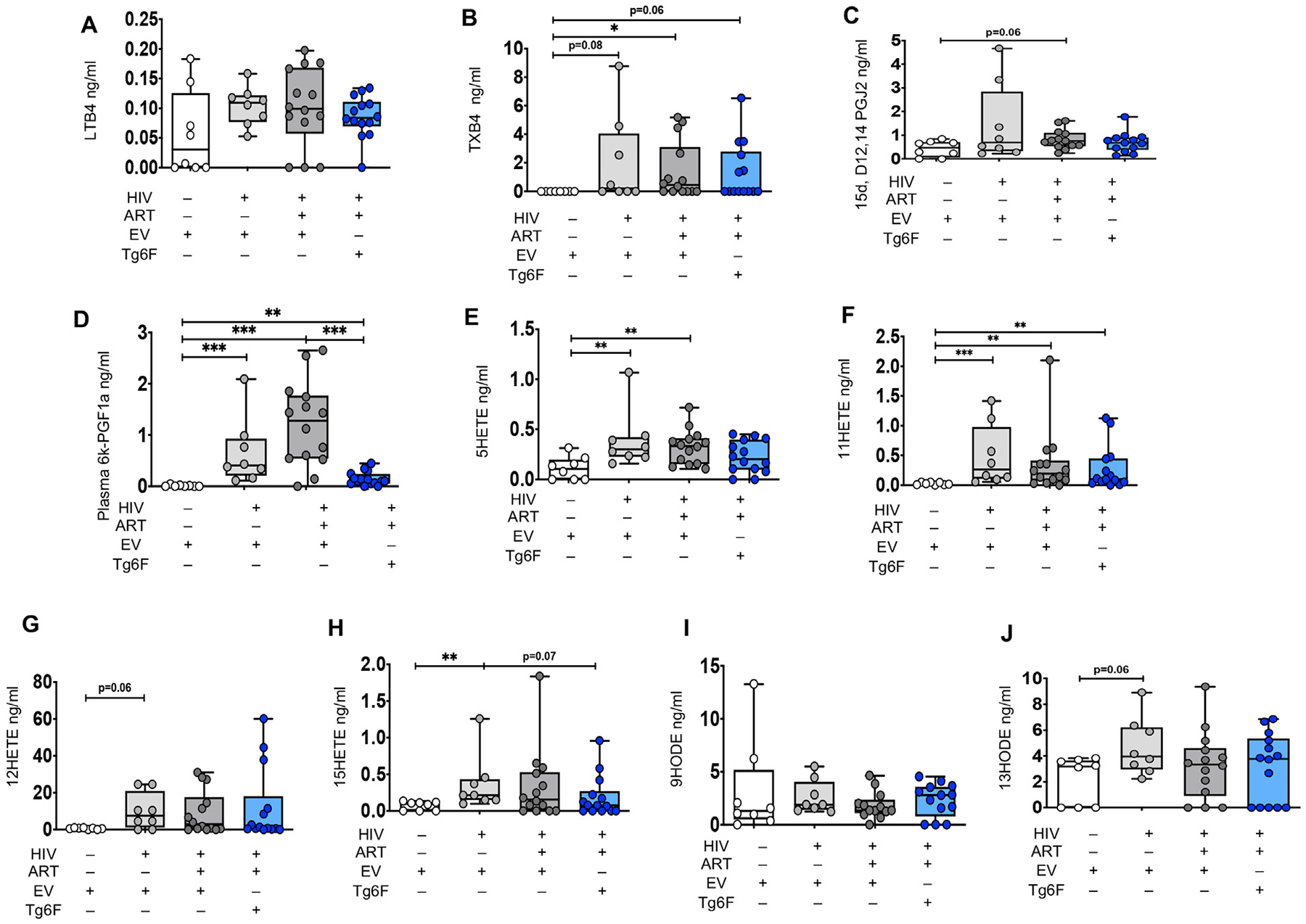
Impact of HIV, ART and Tg6F on plasma bioactive lipids of the Arachidonic acid pathway in TKO humanized mouse model of chronic treated HIV infection. TKO BLT mice (*n* = 45) were constructed, infected with HIV and treated with ART, control transgenic tomato concentrate (EV) or Tg6F as described in Methods. Plasma bioactive lipids of the Arachidonic acid (AA) (A–J), were determined by multiple reaction monitoring (MRM) Liquid Chromatography with tandem mass spectrometry (LC-MS-MS) as described in Methods. Data represent box and whiskers with minimum, median and maximum values of leukotriene B4 (LTB4) (A), thromboxane B2 (TXB2) (B), 15-deoxy-Δ12,14-Prostaglandin J2 (15d-D12,14PGJ2) (C), 6-keto-prostaglandin F1α (6kPGF1α) (D), 5-hydroxyeicosatetraenoic acid (5-HETE) (E) 11-hydroxyeicosatetraenoic acid (11-HETE) (F), 12-hydroxyeicosatetraenoic acid (12-HETE) (G), 15-hydroxyeicosatetraenoic acid (15-HETE) (H), 9-hydroxyoctadecadienoic acid (9HODE) (I), 13-hydroxyoctadecadienoic acid (13HODE) (J). The Mann-Whitney test was used to compare 2 groups (*n* = 8–15 mice per group) (*P < 0.05, **P < 0.01, ***P < 0.001).

**Fig. 3.**
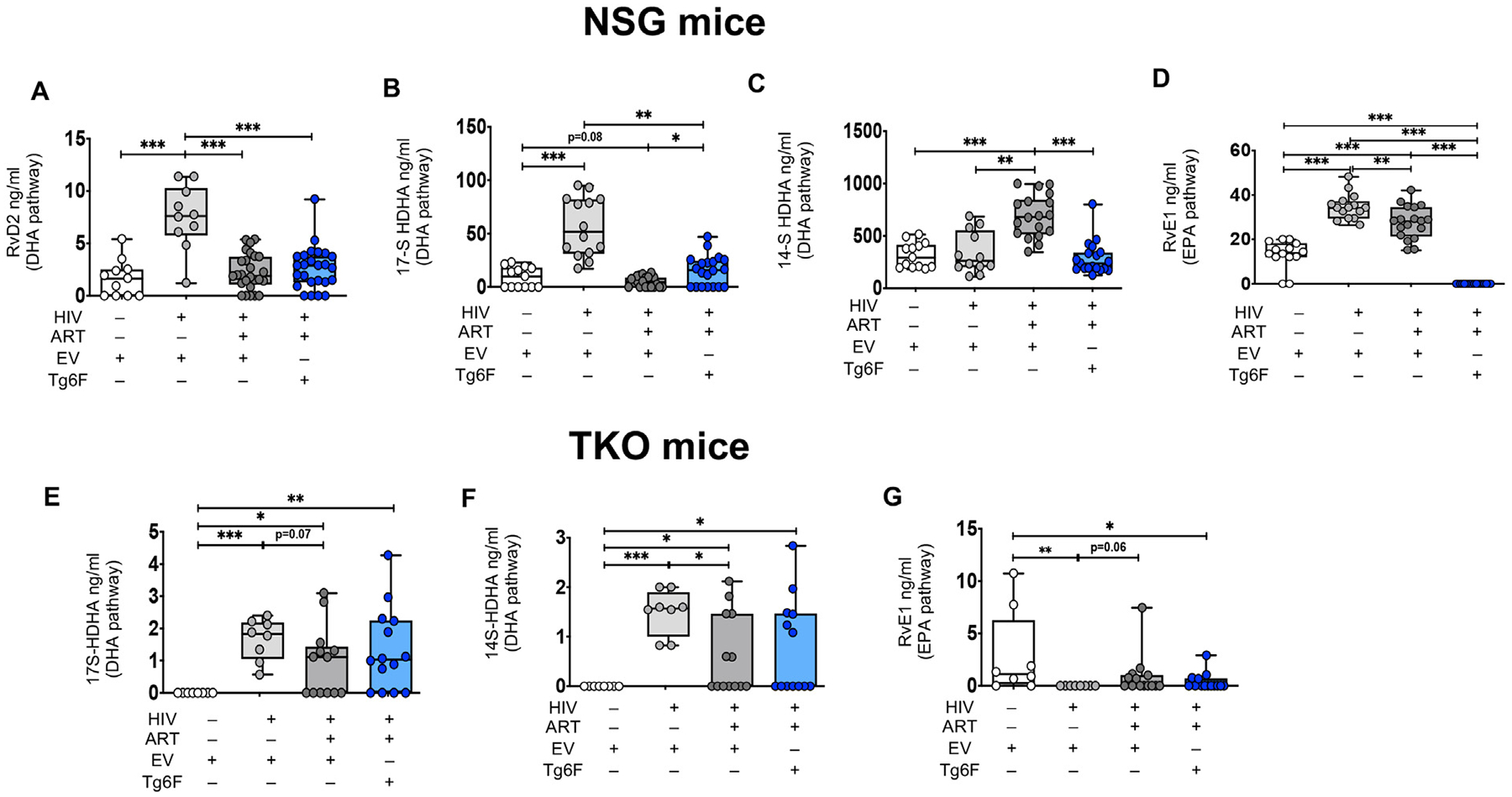
Tg6F attenuates alterations in plasma bioactive lipids of non-Arachidonic acid pathways driven by HIV and/or ART in NSG humanized mouse model of chronic treated HIV infection. NSG (n = 64) and TKO (n = 45) BLT mice were constructed, infected with HIV and treated with ART, control transgenic tomato concentrate (EV) or Tg6F as described in Methods. Plasma bioactive lipids of the Docosahexaenoic acid (DHA) and Eicosapentaenoic acid (EPA) pathways were determined by multiple reaction monitoring (MRM) Liquid Chromatography with tandem mass spectrometry (LC-MS-MS) in NSG (A–D) and TKO (E–G) BLT mice as described in Methods (A–G). Data represent box and whiskers with minimum, median and maximum values of plasma resolvin D2 (RvD2) (A), 17S-hydroxydocosahexaenoic acid (17SHDHA) (B, E), 14S-hydroxydocosahexaenoic acid (14SHDHA) (C, F), resolvin E1 (RvE1) (D, G) in NSG (A–D) and TKO (E–G) BLT mice. The Mann-Whitney test was used to compare 2 groups (*n* = 8–22 mice per group) (*p < 0.05, **p < 0.01, ***p < 0.001).

**Fig. 4.**
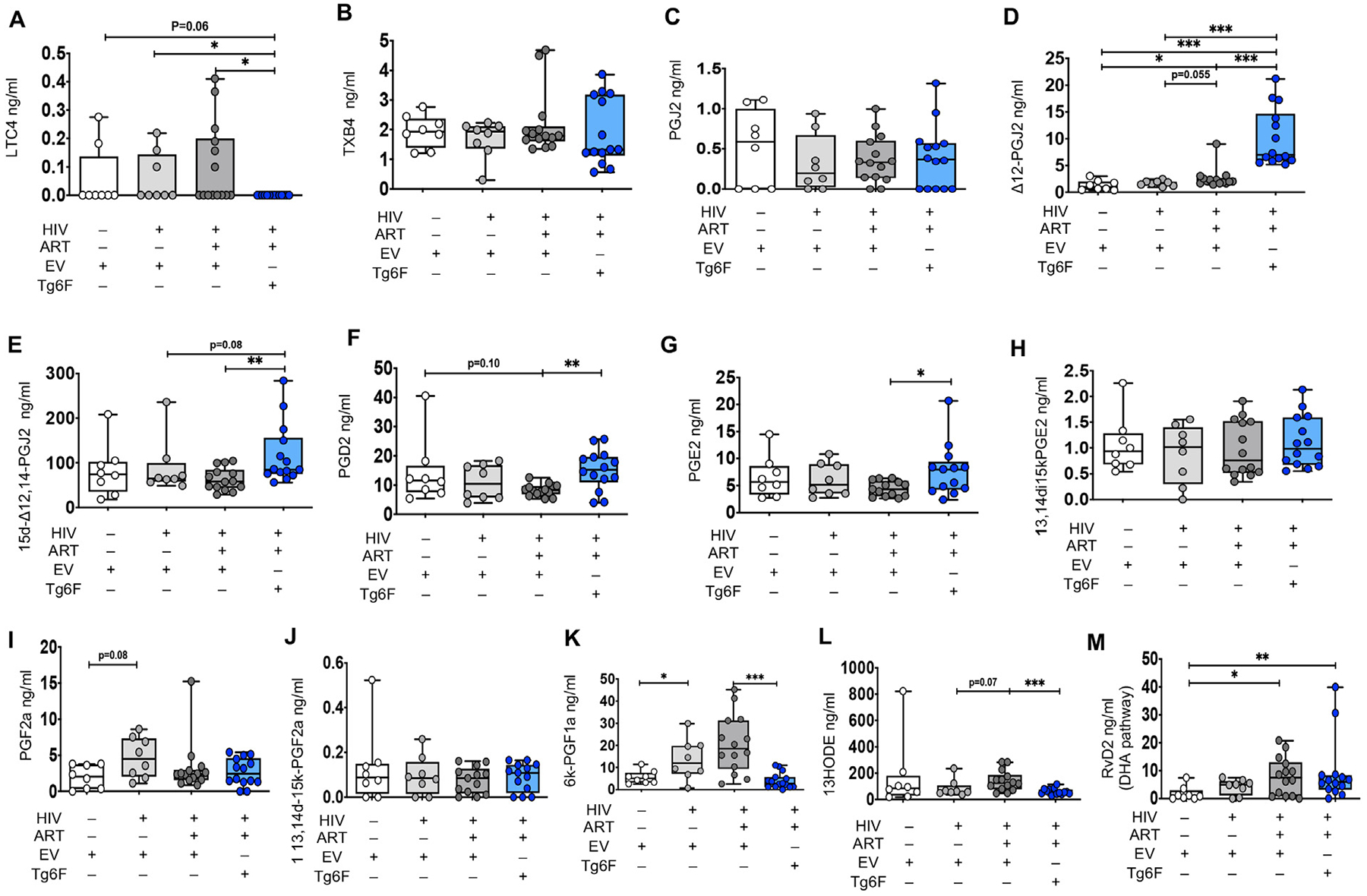
Impact of HIV, ART and Tg6F on gut bioactive lipids in TKO humanized mouse model of chronic treated HIV infection. TKO BLT mice (n = 45) were constructed, infected with HIV and treated with ART, control transgenic tomato concentrate (EV) or Tg6F as described in Methods. Plasma bioactive lipids of the Arachidonic acid (AA) (A–K) and Docosahexaenoic acid (DHA), (L), pathways were determined by multiple reaction monitoring (MRM) Liquid Chromatography with tandem mass spectrometry (LC-MS-MS) as described in Methods. Data represent box and whiskers with minimum, median and maximum values of leukotriene C4 (LTCB4) (A), thromboxane B2 (TXB2) (B), Prostaglandin J2 (PGJ2) (C), Δ12-Prostaglandin J2 (Δ12-PGJ2) (D), 15-deoxy-Δ12,14-Prostaglandin J2 (15d-Δ12,14-PGJ2) (E), Prostaglandin D2 (PGD2) (F), Prostaglandin E2 (PGE2) (G), 13, 14-dihydro-15-keto prostaglandin E2 (13,14dihydro15ketoPGE2) (H), Prostaglandin F2a (PGF2a) (I), 13,14dihydro15ketoPGF2α (13,14d-15k-PGF2a) (J), 6-keto-prostaglandin F1α (6kPGF1α) (K), 13-hydroxyoctadecadienoic acid (13HODE) (L), resolvin D2 (RvD2) (M) The Mann-Whitney test was used to compare 2 groups (*n* = 8–15 mice per group) (*p < 0.05, **p < 0.01, ***p < 0.001).

**Fig. 5.**
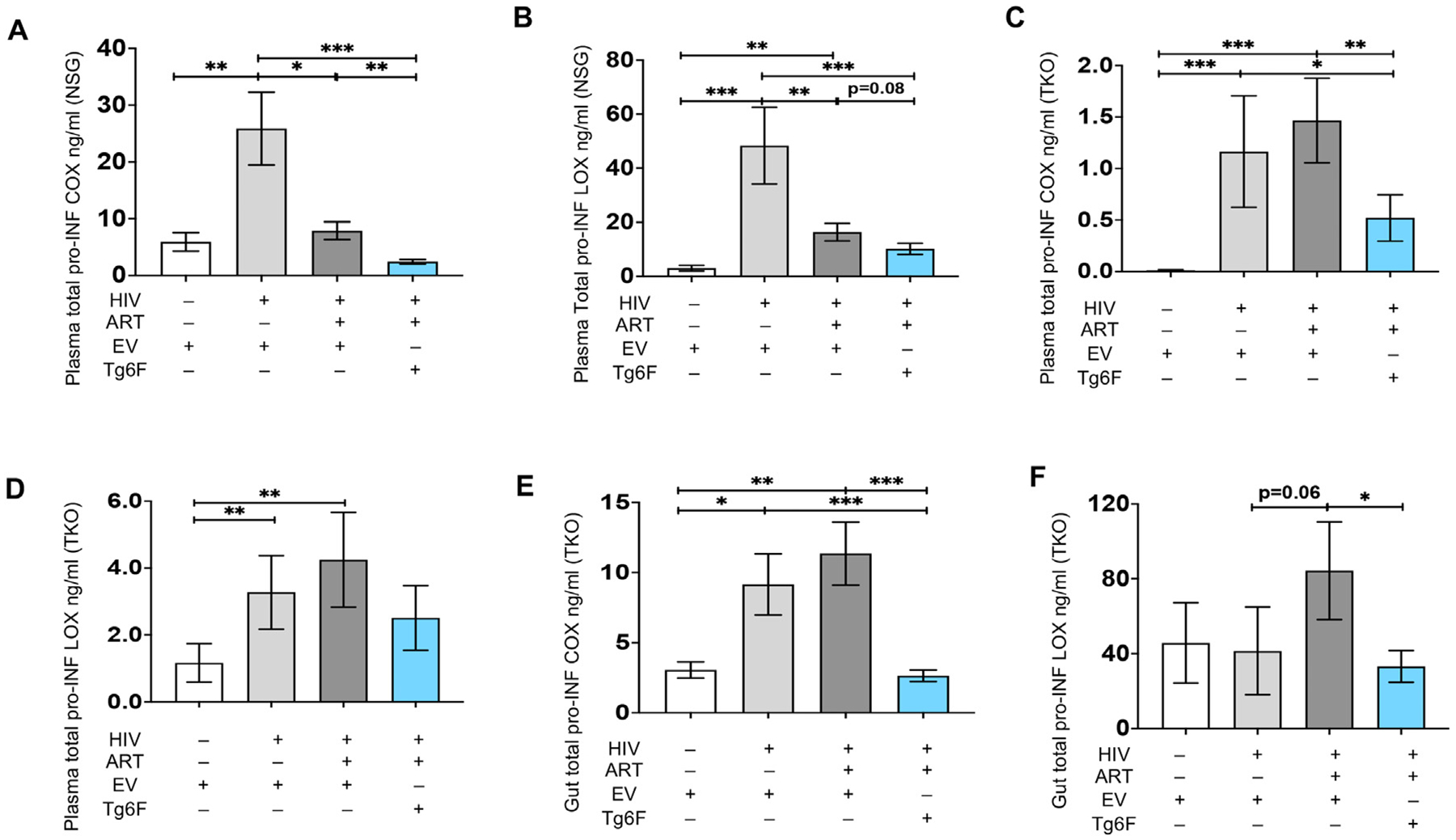
Impact of HIV, ART and Tg6F on plasma and gut bioactive lipids of the cyclooxygenase and lipooxygenase pathways in humanized mouse models of chronic treated HIV infection. TKO C57 (n = 45) and NSG (n = 64) humanized mice were constructed, infected and treated with ART, control transgenic tomato concentrate (EV) or Tg6F as described in Methods. Whole blood and small intestine from each mouse were collected, plasma and tissue homogenates were prepared and murine plasma and gut total proinflammatory bioactive lipid mediators (Total pro-INF) of the cyclooxygenase (COX) pathway (Total pro-INF COX) (TXB2, PGF2α, 6kPGF1α, 11HETE) and the lipooxygenase (LOX) pathway (Total pro-INF LOX) (LTB4, 6trans-12epi-LTB4, 5HETE, 12HETE, 15HETE, 5-oxoETE, 9HODE, 13HODE) were determined in plasma of NSG and TKO BLT mice and homogenate of small intestine from TKO BLT mice by a multiple reaction monitoring liquid chromatography with tandem mass spectrometry (LC-MS-MS) method. Data represent mean ± SEM of plasma total pro-INF COX lipids in NSG mice (A), plasma total pro-INF LOX lipids in NSG mice (B), plasma total pro-INF COX lipids in TKO mice (C), plasma total pro-INF LOX lipids in TKO mice (D), gut total pro-INF COX lipids in TKO mice (E), gut total pro-INF LOX lipids in TKO mice (F). The Mann-Whitney test was used to compare 2 groups (*n* = 8–22 mice per group) (*p < 0.05, **p < 0.01, ***p < 0.001).

We have previously validated a LC-MS-MS method to measure a panel of proinflammatory, lipid mediators (see Supplemental Methods for details) [11]. The method quantitatively evaluates most of the bioactive signaling metabolites of AA, Docosahexaenoic acid (DHA) and Eicosapentaenoic acid (EPA) in the cyclooxygenase (COX) and lipoxygenase (LOX) pathways, together with pathway markers and stable end products [11]. We determined the levels of the above mediators in the proximal small intestine (SI) (where Tg6F mostly acts [11,13,14]) and plasma of TKO and NSG BLT mice. Out of 39 bioactive lipid metabolites (Supplemental material), we consistently detected 15 in plasma *and* gut tissue in *both* BLT mouse models (Figs. 1–5). To dissect the direct impact of HIV-1 on formation of bioactive lipids in blood and gut tissue, we compared levels of bioactive lipids between HIV-1 infected (HIV+) and uninfected BLT mice. Compared to uninfected NSG BLT mice, HIV+ BLT mice had increased plasma levels of AA lipids lipoxin A4 (LXA4), leukotriene B4 (LTB4), 6trans-12epi-LTB4, thromboxane B2 (TXB2), prostaglandin D2 (PGD2), PGE2, 15keto-PGE2, PGF2α, 6-keto-PGF1α (6kPGF1α), (11-hydroxyeicosatetraenoic acid (11-HETE), 12-HETE, 15-HETE and 5-oxoeicosatetraenoic acid (5-oxoETE) (Fig. 1A, C–N) and DHA lipids RvD2, 17S-hydroxydocosahexaenoic acid (17SHDHA), 14SHDHA (Fig. 3A–C) and RvE1 (Fig. 3D). Compared to uninfected TKO mice, HIV+ BLT mice had increased plasma levels of AA lipids 6kPGF1α, 5-HETE, 11-HETE and 15-HETE, tended (0.05 < *p* < 0.10) to have increased plasma levels of TXB4, 12-HETE and 13-hydroxyoctadecadienoic acid (13HODE) (Fig. 2A–J), had increased 17SHDHA and 14SHDHA (Fig. 3E, F) and reduced RvE1 Fig. 3G). HIV-1 infection did not impact plasma levels of other lipids in both NSG (Fig. 1B) and TKO mice.

ApoA-I mimetic peptides work mostly in the gut to attenuate systemic inflammation [15]. A large amount of prostanoids are present in the gut tissue [19]. Given that gut barrier dysfunction drives systemic inflammation in chronic treated HIV, we then determined the impact of HIV-1 infection on intestinal bioactive lipids in GVHD resistant TKO BLT mice with chronic treated HIV. In tissue homogenates from small intestine of TKO BLT mice, compared to the uninfected TKO BLT mice, HIV + TKO BLT mice had increased gut 6kPGF1α (Fig. 4K), tended (*p* = 0.08) to have increased PGF2α (Fig. 4I) and had similar levels of all other detectable AA lipids (Fig. 4A–H, J, L) and RvD2 (Fig. 4M). Thus, in independent humanized mouse models of HIV-1 infection and in both plasma and small intestine tissue, HIV-1 directly and consistently increased production of bioactive AA lipids.

### 3.2. HIV-1 increases plasma and gut levels of bioactive lipids of the COX pathway in vivo

To further characterize the impact of HIV-1 on blood and gut bioactive lipids, we assessed total proinflammatory lipid mediators of cyclooxygenase (COX) (TXB2, PGF2α, 6kPGF1α, 11HETE) (total pro-INF COX) and the lipooxygenase (LOX) pathway (LTB4, 6trans-12epi-LTB4, 5HETE, 12HETE, 15HETE, 5-oxoETE, 9HODE, 13HODE (total pro-INF LOX)) as previously established [11]. In both NSG and TKO mice and compared to uninfected mice, HIV+ BLT mice had a major increase (at least 5-fold) in plasma levels of pro-INF COX lipids (Fig. 5A, C) and increased plasma levels of pro-INF LOX lipids (Fig. 5B, D). Compared to uninfected TKO mice, HIV+ TKO BLT mice had increased gut pro-INF COX lipids (Fig. 5E) but similar levels of gut pro-INF LOX lipids (Fig. 5F). HIV-1 infection induced a major increase (approximately 4-fold) in gut pro-INF COX lipids in HIV+ TKO BLT mice compared to uninfected mice (Fig. 5E). Thus, in independent humanized mouse models of HIV-1 infection, HIV-1 induced a major increase in plasma and gut levels of bioactive COX lipids.

### 3.3. Impact of ART on HIV-1-induced increase in plasma and gut levels of bioactive lipids in vivo

We also assessed the impact of potent ART on plasma and gut bioactive lipids in mouse models of chronic treated HIV. Compared to HIV+ NSG mice, HIV-1 infected/ART-treated (HIV+/ART+) NSG BLT mice had reduced plasma levels of the AA lipids 6trans-12epi-LTB4, TXB2, PGD2, 15keto-PGE2, 11-HETE, 12-HETE, 15-HETE, 5-oxoETE (Fig. 1D, E, F, H, K, L, M, N), reduced plasma levels of the DHA lipids RvD2, 17SHDHA (Fig. 3A, B) and the RvE1 EPA lipid (Fig. 3D) and increased plasma levels of 14SHDHA (Fig. 3C). Compared to HIV+ TKO BLT mice, HIV+/ART+ TKO BLT mice had reduced plasma levels of 14SHDHA (Fig. 3F), tended to have reduced plasma levels of 17SHDHA (Fig. 3E) (*p* = 0.07) and tended (*p* = 0.06) to have increased plasma levels of RvE1 (Fig. 3G), gut levels of Δ12-PGJ2 (p = 0.06) (Fig. 4D) and 13HODE (Fig. 4L). In both NSG (Fig. 1A, B, C, G, I, J) and TKO (Fig. 2A–J) HIV+/ART+ BLT mice, ART did not impact other detectable plasma and gut AA lipids (Fig. 4A–C, E–K, M). Compared to HIV+ NSG BLT mice, HIV +/ART+ NSG BLT mice had reduced plasma levels of pro-INF COX (Fig. 5A) and pro-INF LOX (Fig. 5B) lipids. In contrast to GVHD-prone NSG BLT mice, compared to HIV+ TKO BLT mice, HIV+/ART+ TKO BLT mice had similar levels of plasma pro-INF COX (Fig. 5C), pro-INF LOX (Fig. 5D) and gut pro-INF COX (Fig. 5E) lipids and tended to have increased gut pro-INF LOX lipids (Fig. 5F) (p = 0.06). Overall, in both blood and intestine in independent mouse models of HIV-1 infection, ART did not consistently attenuate HIV-1-induced increases in plasma and intestinal levels of bioactive lipids.

### 3.4. Protein levels of COX-2 are increased in intestinal cells in BLT models of HIV

**Fig. 6.**
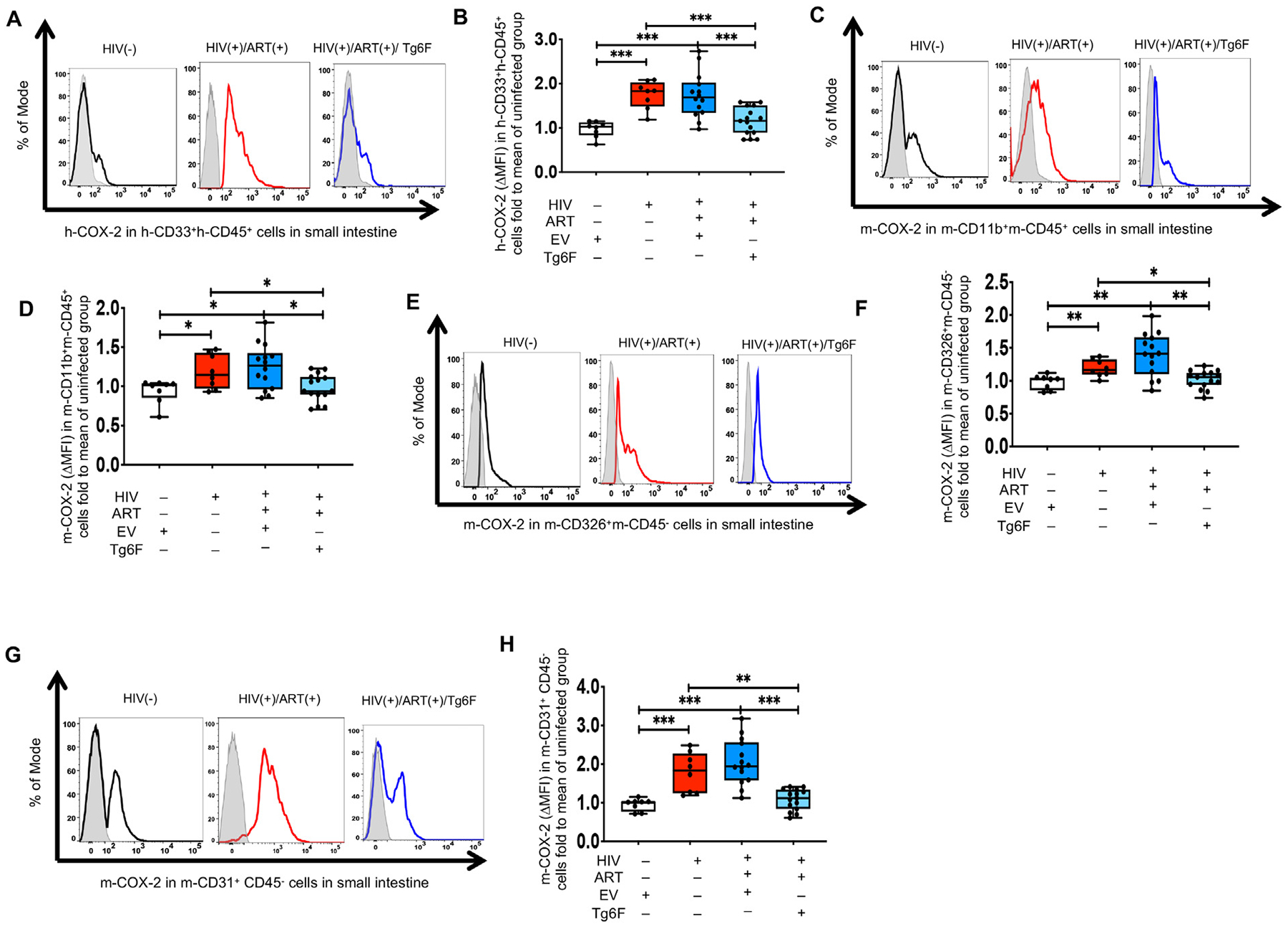
Tg6F attenuates increases in protein levels of COX-2 in intestinal cells of humanized mice with treated HIV infection. TKO C57 (n = 45) humanized mice were constructed, infected with HIV and treated with antiretroviral therapy (ART) and control transgenic tomato concentrate (EV) and/or oral ApoA-I mimetic peptide 6F in the form of transgenic tomato (Tg6F). Small intestine was harvested after 16 weeks of HIV-1 infection. Single cell suspension from gut was prepared and cellular protein levels of human (h-) and murine (m-) cyclooxygenase 2 (COX-2) were determined by flow cytometry in intestinal cells. A. Representative data of change in protein expression of human COX-2 [shift in median fluorescence intensity (ΔMFI) of a positive cell population compared to a negative cell population (fluorescence minus one negative control for staining shown in light filled grey histogram)] in human h-CD33^+^h-CD45^+^ viable single myeloid immune cells are shown. B. Summary of data for (A). C. Representative data of ΔMFI of murine COX-2 in murine CD11b^+^CD45^+^ myeloid immune cells are shown. D. Summary of data for (C). E. Representative data of ΔMFI of murine COX-2 in murine CD326^+^CD45^−^ epithelial cells are shown. F. Summary of data for (E). G. Representative data of ΔMFI of murine ADAM17 in murine CD31^+^CD45^−^ endothelial cells are shown. H. Summary of data for (G). Data represent box and whiskers with minimum, median and maximum values and were normalized against the mean value of the uninfected group. Datapoints represent mean of at least 2 experimental replicates per mouse. The Kruskal Wallis was used to compared >2 groups and the Mann-Whitney test was used to compare 2 groups (*n* = 8–15 mice per group) (*p < 0.05, **p < 0.01, ***p < 0.001).

Our data showed in both HIV+/ART+TKO and NSG BLT mice that HIV-1 infection induced formation of *murine* bioactive lipids and especially COX-2 bioactive lipids. COX-2 mediates the production of bioactive lipids by macrophages, endothelial cells and/or enterocytes in response to bacterial products like LPS [8]. Given that HIV-1 infects only human immune cells in BLT mice, we hypothesized that HIV-1 infection through gut barrier dysfunction, bacterial translocation and increased LPS in humanized mice [15], induces COX-2 in human myeloid cells and in murine myeloid, endothelial and epithelial cells in gut tissue. To validate this hypothesis, we used flow cytometry to study COX-2 at the single cell level in small intestine tissue from GVHD-resistant TKO BLT mice. Examples of gating of human and murine cells in humanized mice have previously been shown [15]. Compared to uninfected mice, chronic HIV increased the protein expression (median fluorescence intensity; MFI) of hCOX-2 in h-CD33^+^h-CD45^+^ gut myeloid cells (Fig. 6A, B), mCOX-2 in murine CD11b^+^CD45^+^ gut myeloid immune cells (Fig. 6C, D), in murine CD326^+^CD45^−^ epithelial cells (Fig. 6E, F) and murine CD31^+^CD45^−^ endothelial cells (Fig. 6G, H). The largest impact of HIV-1 infection was observed in murine endothelial cells (Fig. 6G, H). Importantly, potent ART did not reduce COX-2 in gut human myeloid cells (Fig. 6A, B) and in murine gut myeloid immune cells (Fig. 6C, D), epithelial cells (Fig. 6E, F) and endothelial cells (Fig. 6G, H). Collectively, our data demonstrate that consistent with the increase in blood and intestinal levels of COX-2 lipids, protein levels of COX-2 are also increased in intestinal cells (myeloid, endothelial and epithelial cells) in BLT models of chronic treated HIV.

### 3.5. Tg6F attenuates plasma and gut levels of bioactive lipids and intestinal levels of COX-2 that are not altered by potent ART in HIV-1 infection

We hypothesized that an oral intervention like apoA-I mimetic peptides that act in the gut to directly bind microbial products like LPS and bioactive lipids that also attenuate aberrant COX activity in inflammatory conditions [11], may attenuate production of proinflammatory bioactive lipids in chronic treated HIV. Therefore we treated with Tg6F administered in a chow diet (0.06% by weight) to HIV+/ART+TKO BLT mice that had suppressed plasma viremia after 4 weeks of potent ART (HIV+/ART+/Tg6F + TKO group), as previously described [15]. The uninfected, HIV+ and HIV+/ART+ mice were all fed with chow diet that contained control transgenic tomato concentrate (EV) to ensure that the effectiveness of Tg6F is due to the presence of 6F, which is not present in EV [13,14]. We also cotreated HIV+/ART+ NSG BLT mice with potent ART for 10 weeks with a chow diet containing 0.06% Tg6F (wt/wt) as previously described [15]. We previously demonstrated that Tg6F did not impact plasma viremia in NSG BLT mice [15]. To assess if Tg6F might complement ART after suppression of HIV-1 viremia, and to identify potential *additional* therapeutic benefits of Tg6F, we focused on comparisons between the HIV+/ART+/Tg6F+ mice and the HIV+/ART+ BLT mice (not the HIV+ BLT mice).

First, we assessed the impact of Tg6F on blood bioactive lipids in BLT models of chronic treated HIV. Compared to HIV+/ART+ NSG BLT mice, the HIV+/ART+/Tg6F+ mice expressed lower levels of the AA lipids LXA4, 15epi-LXA4, LTB4, TXB2, PGF2α, 6kPGF1α, 12-HETE (Fig. 1A–C, E, I, J, L), 17SHDHA (Fig. 3C) and RvE1(Fig. 3D), increased plasma levels of PGD2 (Fig. 1F) and 17SHDHA (Fig. 3B) and similar plasma levels of other AA lipids (Fig. 1D, G, H, K, M, N) and RvD2 (Fig. 3A). Compared to HIV+/ART+ TKO BLT mice, the HIV+/ART+/Tg6F+ mice expressed lower levels of plasma 6kPGF1α (Fig. 2D) but had similar plasma levels of all other AA lipids (Fig. 2A–C, E–J), 17SHDHA, 14SHDHA and RvE1 (Fig. 3E–G). Compared to HIV+/ART+ NSG BLT mice, HIV+/ART+/Tg6F+ ^+^ NSG BLT mice had reduced plasma levels of pro-INF COX (Fig. 5A) and tended (*p* = 0.08) to have reduced pro-INF LOX (Fig. 5B) bioactive lipids. Compared to HIV+/ART+ TKO BLT mice, HIV+/ART+/Tg6F+ TKO BLT mice had reduced levels of plasma pro-INF COX (Fig. 5C) and similar levels of plasma pro-INF LOX (Fig. 5D).

Compared to HIV+/ART+ TKO BLT mice, HIV+/ART+/Tg6F+ TKO BLT mice had reduced gut levels of LTC4 (Fig. 4A), 6kPGF1α (Fig. 4K), 13HODE (Fig. 4L), pro-INF COX (Fig. 5E) and LOX (Fig. 5F) lipids, increased gut levels of Δ12-PGJ2 (Fig. 4D), 15d-Δ12,14-PGJ2 (Fig. 4E), PGD2 (Fig. 4F) and PGE2 (Fig. 4G) and similar gut levels of all other detectable bioactive lipids of the AA pathway (Fig. 4B, C, H, I, J) and RvD2 (Fig. 4M). Importantly, unlike potent ART, Tg6F reduced HIV-1-induced increase in protein expression of hCOX-2 in gut human myeloid cells (Fig. 6A, B), in murine gut myeloid immune cells (Fig. 6C, D), in murine epithelial cells (Fig. 6E, F) and murine endothelial cells (Fig. 6G, H).

Thus, in independent BLT models and unlike ART and consistently with our prior data that apoA-I mimetic peptide reduce proinflammatory bioactive lipids and aberrant COX-2 activity in mouse models of inflammatory bowel disease [11], Tg6F therapy for up to 14 weeks attenuated HIV-induced increases in plasma and gut biolipids and intestinal COX-2 protein levels.

### 3.6. 4F and 6F attenuate ex vivo intestinal COX-2 protein levels in gut explants of HIV infected ART-treated participants

**Fig. 7.**
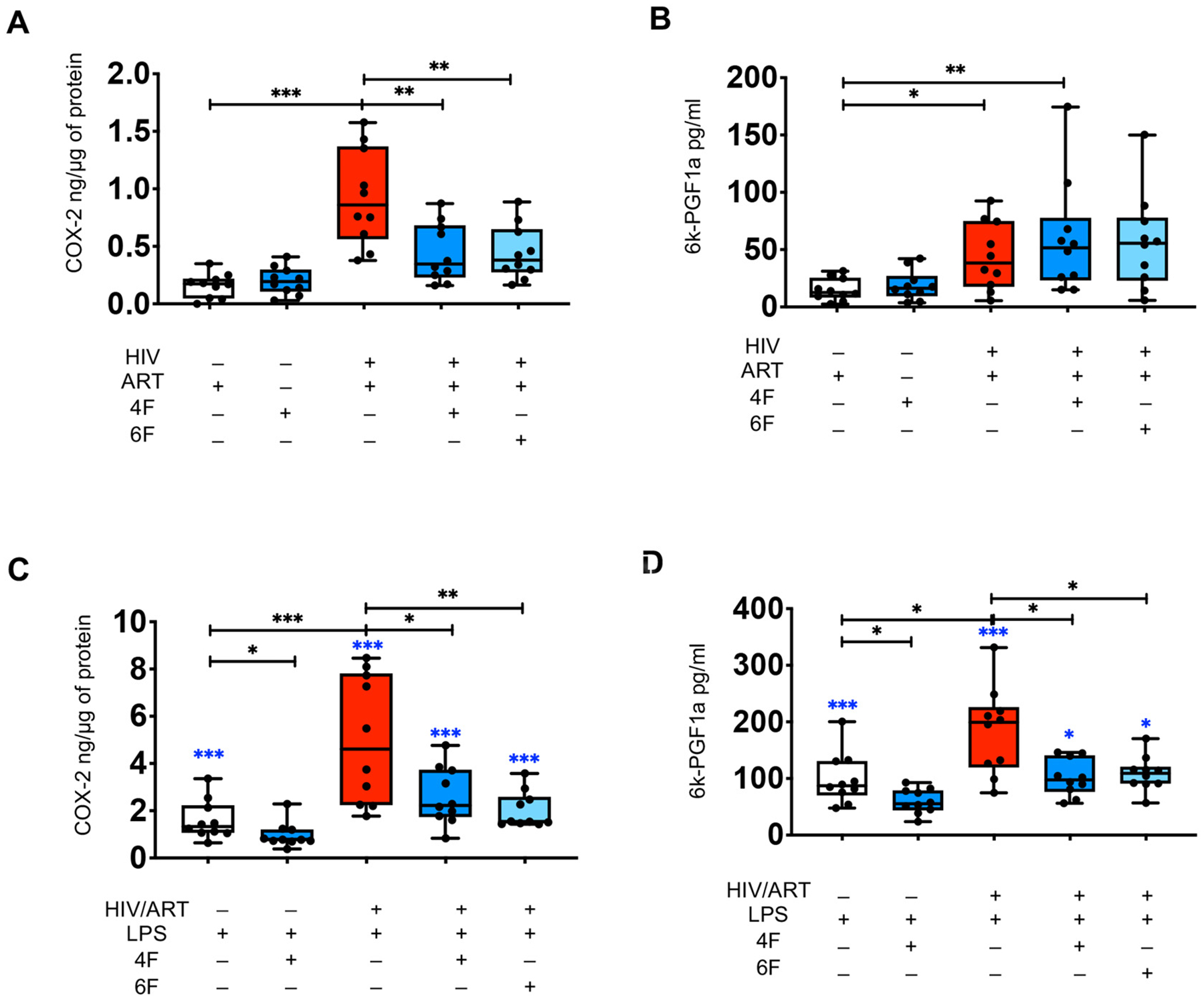
ApoA-I mimetic peptides attenuate *ex vivo* aberrant increase in proteins levels of cyclooxygenase 2 (COX-2) and associated production of bioactive lipids that reflect prostacyclin synthesis in gut explants of HIV infected participants. Gut biopsies were obtained from uninfected (*n* = 10) and HIV infected participants on potent antiretroviral therapy (ART) (n = 10) and gut explants were treated with 4F or 6F apoA-I mimetic peptides or sham peptide and/or endotoxin (LPS) for 72 h as in methods. Gut biopsies from the same participants were used for experiments with treatments. Protein levels of cyclooxygenase 2 (COX-2) (ng/μg of total protein) were determined by ELISA in tissue homogenates from gut biopsies of uninfected and HIV infected participants treated as shown. Supernatants were collected at 72 h and secreted levels of 6-keto Prostaglandin F_1α_ (6 k-PGF1a) (pg/ml), a surrogate for prostacyclin synthesis, were determined in culture supernatants from gut biopsies by ELISA. A, C. Protein levels of COX-2 in gut tissue lysates treated as shown. B, D. Secreted levels of 6 k-PGF1a in culture supernatants from gut biopsies treated at shown. Data represent box and whiskers with minimum, median and maximum values. Datapoints represent mean of 3 gut biopsies per participant. The Mann-Whitney test was used to compare 2 groups (n = 10 per group). The comparisons of the LPS-treated groups (C, D) with the respective group that was not treated with LPS (A, B) are shown in blue asterisk and the Mann-Whitney test was also used to compare 2 groups (n = 10 per group)(*p < 0.05, **p < 0.01, ***p < 0.001).

BLT mice have known limitations [15] and formation of bioactive lipids may differ between different mouse strains and humans. To address these limitations, we also used colon tissues of uninfected (*n* = 10) and HIV infected (n = 10) ART-treated 50–60 years old white men without clinical morbidity to further validate our results that apoA-I mimetic peptides attenuate increased COX-2 protein levels and its associated production of 6kPGF1α, a major product of the endothelium, in chronic treated HIV. We used ELISA to measure *ex vivo* after 72 h, total intestinal COX-2 protein levels in gut tissue homogenates from gut explants and secreted 6kPGF1α in supernatants of gut explants from HIV infected and uninfected participants. Compared to uninfected participants, gut explants of HIV+/ART+ participants had higher protein levels of intestinal COX-2 (Fig. 7A) and higher levels of 6kPGF1α that was secreted by gut explants (Fig. 7B). 4F did not reduce *ex vivo* intestinal COX-2 levels (Fig. 7A) and secretion of 6kPGF1α (Fig. 7B) in gut explants from uninfected participants (Fig. 7A). Both 4F and 6F, did not reduce *ex vivo* secretion of 6kPGF1α by gut explants from HIV+/ART+ participants (Fig. 7B). In contrast, both 4F and 6F, similarly and consistently reduced *ex vivo* COX-2 intestinal protein levels in gut explants from HIV+/ART+ participants (Fig. 7A). Thus, our data confirmed that COX-2 intestinal levels are increased in gut tissues from HIV+/ART+ compared to uninfected participants and that apoA-I mimetic peptides attenuate *ex vivo* aberrant increase of COX-2 protein levels in gut tissues from HIV+/ART+ participants.

### 3.7. 4F and 6F attenuate ex vivo proinflammatory effect of LPS on intestinal COX-2 levels and associated prostacyclin activity in chronic treated HIV

We previously demonstrated that Tg6F reduced plasma LPS in BLT mouse models of chronic treated HIV [15]. ApoA-I mimetics reduce proinflammatory activity of LPS *in vivo* [11], an instigator of COX-2 and prostacyclin [20–22]. We also showed that HIV+/ART+ BLT mice had increased intestinal levels of COX-2 and 6kPGF1α *in vivo*. We hypothesized that the inhibitory effect of apoA-I mimetic peptides on COX-2 is mediated through LPS. To confirm our hypothesis, we tested whether LPS increases *ex vivo* protein levels of COX-2 and *ex vivo* secretion of 6kPGF1α from colon tissues of uninfected and HIV+/ART+ men. Gut explants of both uninfected and HIV+/ART+ participants treated with LPS had higher protein levels of intestinal COX-2 (Fig. 7A, C) and secreted levels of 6kPGF1α (Fig. 7B, D) compared to vehicle controls. In contrast to vehicle controls (Fig. 7A), 4F reduced *ex vivo* intestinal COX-2(Fig. 7C) and secreted levels of 6kPGF1α (Fig. 7D) in gut explants from uninfected participants treated with LPS. Both 4F and 6F, similarly and consistently reduced *ex vivo* intestinal COX-2 (Fig. 7C) and secreted 6kPGF1α (Fig. 7D) in gut explants from HIV+/ART+ participants treated with LPS. Collectively, our data showed that 4F and 6F attenuate *ex vivo* proinflammatory effect of LPS on intestinal COX-2 levels and associated prostacyclin activity in chronic treated HIV.

## 4. Discussion

**Fig. 8.**
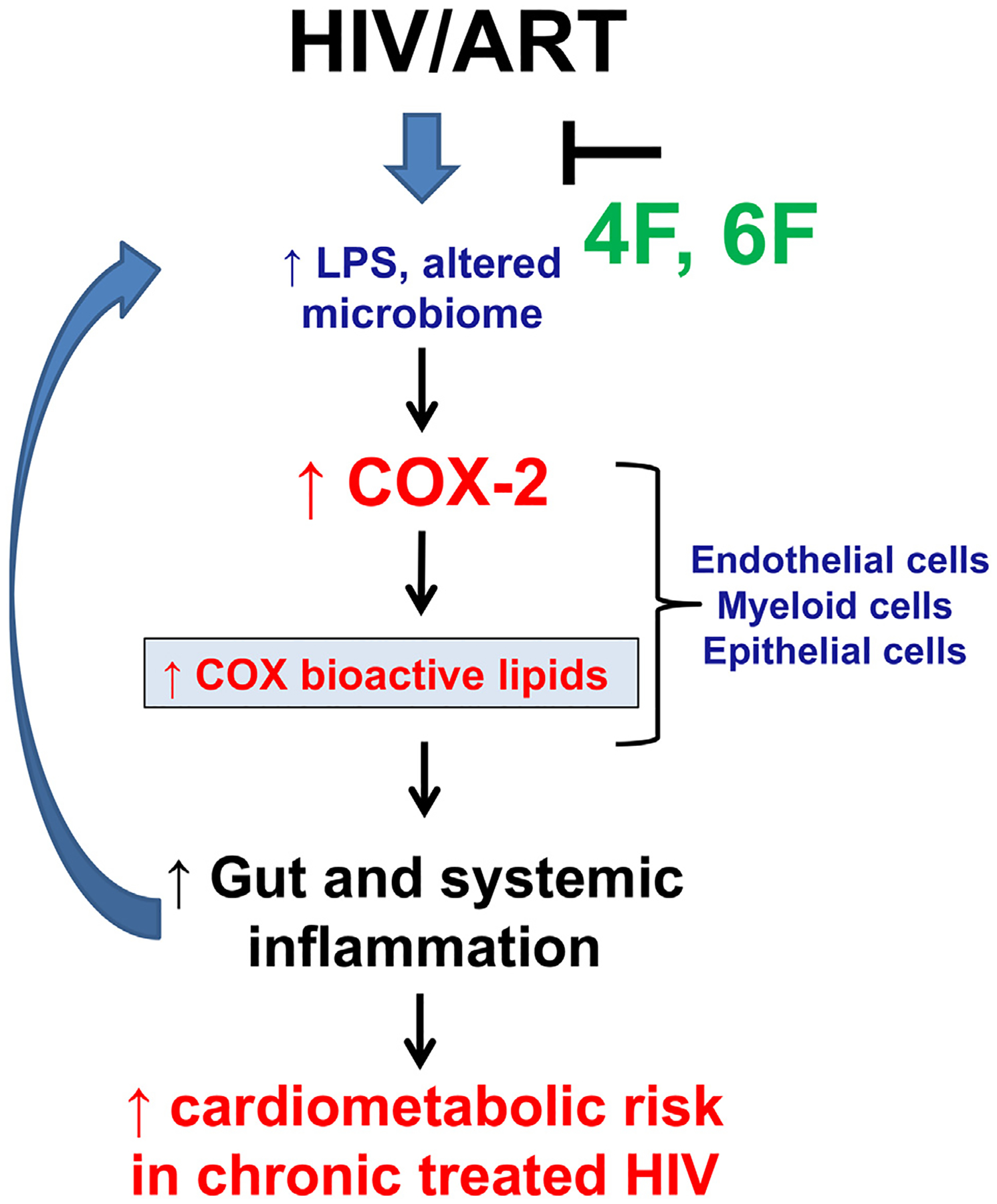
Overall hypothesis. Increased gut barrier dysfunction leads to altered microbiome and increased systemic and intestinal levels of endotoxin (LPS) in chronic treated HIV despite potent antiretroviral therapy (ART). LPS increases protein levels of cyclooxygenase 2 (COX-2) in myeloid, epithelial and endothelial cells in intestine and other tissues of HIV infected persons and induces production of COX proinflammatory bioactive lipids. Increased proinflammatory activity of COX and associated bioactive lipids further drive gut inflammation, gut barrier dysfunction and systemic inflammation and ultimately increase cardiometabolic risk in chronic treated HIV. ApoA-I mimetic peptides (4F, 6F) act mostly in the intestine and reduce levels of bioactive lipids, LPS and microbial translocation, leading to reduction in levels of COX-2 and associated production of bioactive lipids in blood and the intestine. Thus, ApoA-I mimetic peptides show potential to reduce increased gut and systemic inflammation and cardiometabolic risk in chronic treated HIV.

Using independent preclinical approaches with physiologically relevant mouse models of chronic treated HIV and gut explants from HIV infected and uninfected persons, we show that levels of intestinal COX-2 and associated production of proinflammatory COX AA lipids, are increased in blood and intestine in chronic treated HIV. ApoA-I mimetics consistently attenuated intestinal levels of COX-2 and bioactive lipids in models of chronic treated HIV. Tg6F consistently attenuated aberrant production of bioactive lipids of COX, LOX, AA, DHA and EPA pathways in HIV-1 infected mice on potent ART. The component of Tg6F accounting for the beneficial effects on bioactive lipids and aberrant increase in COX-2 in chronic treated HIV is the 6F peptide, since the EV control had no effect on altered bioactive lipids and COX-2. Overall, our results suggest that apoA-I mimetic peptides attenuate the crosstalk between increased gut barrier dysfunction, LPS, COX-2 and associated production of COX-2 lipids in gut tissue that contribute to gut and systemic inflammation and cardiometabolic risk in chronic treated HIV (Fig. 8). Given that Tg6F seems to work exclusively in the gut to reduce gut barrier dysfunction [15] our model (Fig. 8) is proof-of-concept that therapeutic targeting of aberrant increase in levels of intestinal LPS and bioactive lipids and gut barrier dysfunction with apoA-I mimetics may complement ART to favorably impact inflammation and cardiometabolic risk in chronic treated HIV.

Chronic treated HIV is a state of increased bacterial translocation and proinflammatory effects of LPS [1,23]. LPS activates TLR4 and downstream NF-κB to induce the synthesis of proinflammatory cytokines [24]. Notably, COX-2 is implicated in TLR4 and NF-κB signaling in inflammatory diseases and cancer [25,26]. LPS directly upregulates *in vitro* COX-2 levels in immune, epithelial and endothelial cells [20–22]. Notably, in both BLT models, COX-2 was also increased in murine cells that were not infected by HIV suggesting a direct proinflammatory effect of murine LPS that was upregulated in BLT models of chronic treated HIV. Our *ex vivo* studies with gut explants further confirmed that LPS directly induces intestinal levels of COX-2 and associated production of prostacyclin products in chronic treated HIV. This evidence is consistent with prior studies that exposure to microbial products activates macrophages and COX-2 lipids like PGE_2_ [27,28] to alter immune responses to HIV infection [29–31] in both untreated [31] and ART-treated patients [32,33]. Our prior studies showed that apoA-I mimetic peptides reduced LPS and bacterial translocation in mouse models of chronic treated HIV [15] and inflammatory bowel disease [11]. Together with the data presented here and prior human studies that metabolic endotoxemia may contribute to development of cardiometabolic disease [34–36], the use of apoA-I mimetic peptides is suggested as a novel therapeutic strategy to attenuate increased cardiometabolic risk in chronic treated HIV.

COX-2 is a major source of prostanoids in inflammatory conditions and diseases such as cardiovascular disease [8], inflammatory bowel disease [11], obesity and the metabolic syndrome [9]. COX-2 induction has been shown in *all* the cells involved in HIV end organ disease and gut barrier dysfunction, including endothelial cells, epithelial cells, macrophages, and other cell types, in response to different stimuli [8,37]. Similar to other inflammatory states [38], we found that the largest impact of HIV-1 infection on COX-2 levels and associated bioactive lipids was seen in endothelial cells and the prostacyclin derivative 6kPGF1α. Although our studies focused on accessible intestinal tissues and blood COX-2 bioactive lipids, our data suggest systemic upregulation of COX-2 in potentially other tissues in chronic treated HIV. Since aberrant increase in COX-2 activity in metabolically active tissues like adipose tissue and liver may contribute to pathogenesis of cardiometabolic syndrome by alteration of adipokines and insulin sensitivity [9], further studies are needed to establish the understudied COX-2 pathway in HIV as major proinflammatory pathway that drives cardiometabolic disease in chronic treated HIV.

Our overall hypothesis, corroborates evidence that COX-2 is a potential therapeutic target for inflammatory conditions [37] including viral infections (such as COVID-19) [39]. Importantly, an exploratory trial of a COX-2 inhibitor in HIV-1 Infection reduced immune activation and improved T cell functions in HIV-infected patients [31]. Our studies demonstrated an *additional* therapeutic benefit of apoA-I mimetic peptides compared to potent ART on aberrant alterations of bioactive lipids in mouse models of chronic treated HIV. The safety and pharmacokinetics of oral apoA-I mimetic peptides have been tested in humans [40]. Given that non-steroidal anti-inflammatory drugs cannot be taken long term by HIV-1 infected persons (due to toxicity in kidneys and intestine), we propose the first therapeutic strategy that can target COX-2 activity in chronic treated HIV.

Our study has several limitations. BLT mice do not recapitulate pathogenesis of HIV-1 infection due to variable suboptimal engraftment of human immune cells, lack of fully functional human lymphoid tissues, presence of GVHD and non-human microbiome [18,41–43]. Intestinal explants focus only on early signaling in response to different stimuli. Thus, other underlying mechanisms or variables in our preclinical models may confound interpretation of data. Despite the known limitations of BLT mice, we have demonstrated the ability of independent BLT mouse models to support HIV infection, express clinically relevant biomarkers of intestinal and systemic inflammation in the setting of chronic treated HIV and consistently respond to clinically relevant ART [15]. We did not include NSG mice with clinical GVHD and we also studied GVHD-resistant TKO BLT mice [42,43]. Gut explants also do not have limitations of other *ex vivo* intestinal models [44] and *ex vivo* use of 4F in gut explants is the *only* preclinical approach in humans at this time. Finally, our “proof of concept” preclinical studies focused on physiologically relevant mediators of cardiometabolic disease (COX-2, bioactive lipids) rather than cardiometabolic disease *per se*. Further experimental preclinical and clinical studies are needed to establish the role of altered COX-2 activity in cardiometabolic disease *per se* in chronic treated HIV.

## 5. Conclusions

Independent preclinical experimental models support the hypothesis that apoA-I mimetic peptides can attenuate a cycle of increased gut barrier dysfunction, intestinal inflammation, production of bioactive lipids and microbial products that contribute to further inflammation, cardiometabolic risk and morbidity in chronic treated HIV. A future clinical trial in chronic treated HIV may fully assess the impact of oral 4F on the microbiome, gut barrier integrity and key bioactive lipids that drive cardiometabolic risk in chronic treated HIV. These preclinical translational studies increase our understanding of the anti-inflammatory mechanisms of apoA-I mimetic peptides against aberrant COX-2 activity and may advance their use in chronic treated HIV.

## Supplementary Material

Supplemental Material

## Data Availability

All data needed to understand and assess the conclusions of this research are available in the main text and Supplementary materials. Raw datasets supporting the findings of this study are available from the corresponding author on reasonable request. The availability of Tg6F is subject to a material-transfer agreement, which can be requested through the corresponding author.

## Abbreviations:

ART: antiretroviral therapy (ART)
BLT: Bone Marrow/Liver/Thymus mouse
C57BL/6: C57 black 6
EV: a concentrate of transgenic control tomatoes
FTC: Emtricitabine
GVHD: graft *versus* host disease
HIV: Human Immunodeficiency Virus
HDL: High Density Lipoprotein
LC-MS-MS: Liquid Chromatography with tandem mass spectrometry
LPS: lipopolysaccharide or endotoxin
MFI: mean fluorescence intensity
NSG: NOD.Cg-*Prkdc*^*scid*^*Il2rg*^*tm1Wjl*^/SZJ or NOD/SCID/IL2Rγ−/−
PBS: phosphate buffered saline
Tg6F: a concentrate of transgenic tomatoes that express the apoA-1 mimetic peptide 6F
TKO: recombination activating gene 2 (Rag2)γcCD47 triple knockout or *Rag2*−/−*γc*−/−*CD47*−/−
