# Supplementary material for: ApoA-I mimetics favorably impact cyclooxygenase 2 and bioactive lipids that may contribute to cardiometabolic syndrome in chronic treated HIV
Source: Metabolism. Author manuscript; Available in PMC 2022 Jan 31. (PMC8802211; doi:10.1016/j.metabol.2021.154888)
Supplement: Supplemental Material [file NIHMS1773485-supplement-Supplemental_Material.docx]

**Supplemental Material**

**Supplemental Materials and Methods**

**Materials**

The following chemicals were purchased from Cayman Chemicals (Ann Arbor, MI, USA): 18-hydroxyeicosapentaenoic acid (18-HEPE); 6-keto-prostaglandin F1α (6kPGF1α); resolvin E1 (RvE1); resolvin E1 deuterated (RvE1-d4); thromboxane B3 (TXB3); thromboxane B2 (TXB2); thromboxane B2 deuterated (TXB2-d4); prostaglandin E3 (PGE3); 20-hydroxy-leukotriene B4 (LTB4); prostaglandin F2α (PGF2α); prostaglandin E2 (PGE2); prostaglandin E2 deuterated (PGE2-d4); resolvin D3 (RvD3); resolvin D3 deuterated (RvD3-d5); lipoxin B4 (LXB4), prostaglandin D2 (PGD2); prostaglandin D2 deuterated (PGD2-d4); resolvin D2 (RVD2); resolvin D2 deuterated (RvD2-d5); leukotriene C4 (LTC4); leukotriene C4 deuterated (LTC4-d5); 15-keto-prostaglandin E2 (15keto-PGE2); leukotriene E4 (LTE4); lipoxin A4 (LxA4); lipoxin A4 deuterated (LxA4-d5); 15-epi-lipoxin A4 (15epi-LXA4); resolvin D1 (RvD1); resolvin D1 deuterated (RvD1-d5); 13, 14-dihydro-15-keto prostaglandin F2α (13,14-dihydro-15keto- PGF2α); 13, 14-dihydro-15-keto prostaglandin F2α deuterated (13,14-dihydro-15-keto-PGF2α-d4); 13, 14-dihydro-15-keto prostaglandin E2 (13,14-dihydro-15keto-PGE2); 13, 14-dihydro-15-keto prostaglandin E2 deuterated (13,14-dihydro-15-keto-PGE2-d4); resolvin D4 (RvD4); 13, 14-dihydro-15-keto prostaglandin D2 (13,14-dihydro-15keto-PGD2); prostaglandin J2 (PGJ2); Δ12-Prostaglandin J2 (Δ12-PGJ2); 7(S)-maresin (MaR1); 7(S)-maresin deuterated (MaR1-d5); 10(S),17(S)-protectin (PDx); 6-trans-12-epi leukotriene B4 (6trans-12epi-LTB4); resolvin D5 (RvD5); leukotriene B4 (LTB4); leukotriene B4 deuterated (LTB4-d4); 15-deoxy-Δ12,14-Prostaglandin J2 (15d-PGJ2); 15-deoxy-Δ12,14-Prostaglandin J2 deuterated (15d-PGJ2-d4); 13-hydroxyoctadecadienoic acid (13-HODE), 9-HODE, 13-HODE-d4; 15- hydroxyeicosatetraenoic acid (15-HETE) and 12-HETE, 5-HETE, 15-HETE-d8, 12-HETE-d8, 5-HETE-d8, and 11-HETE; 17S-hydroxydocosahexaenoic acid (17S-HDHA) and 14S-HDHA; 5-oxoeicosatetraenoic acid (5-oxoETE); and 5-oxoeicosatetraenoic acid deuterated (5-oxoETE-d7). Precellys ceramic beads were also purchased from Cayman.

The following kits were purchased for immunoassays: mouse COX-2 ELISA (MyBiosource), 6-keto Prostaglandin F1α ELISA (Cayman Chemical).

Flow cytometry reagents including flow cytometry staining buffers and Red Cell Lysis buffer solution were purchased from Biolegend. All antibodies were obtained from Biolegend except for PE anti-COX-2 (clone COX 229), which was obtained from Thermo Scientific. For flow cytometry, single cell suspensions were incubated with the following mouse antibodies: SYTOX Blue (Biolegend), Alexa Fluor® 700 anti-mouse CD45 (clone 30-F11), FITC anti-human CD33 Antibody (clone P67.6), PE anti-human CD45 (clone HI30), PerCP/Cy5.5 anti-mouse CD326 (Ep-CAM, clone G8.8), PE-Texas Red anti-mouse CD11b (clone M1/70).

The following materials were purchased from Thermo Scientific methanol, T-PER™ tissue protein extraction Reagent, sterile 70 μm nylon filter, Lipofectamine 2000 reagent, 293FT cells, Dulbecco’s modified Eagle medium (DMEM), Opti-MEM, Amicon Ultra-15 Centrifugal Filter Unit, fetal bovine serum, 1% penicillin/streptomycin, and 1% non-essential amino acids.

The following materials were purchased from Sigma Aldrich: butylated hydroxytoluene (BHT), Triton-x-100, RIPA buffer, Protease Inhibitor Cocktail, Triglyceride Quantification Colorimetric/Fluorometric Kit. Oasis HLB SPE cartridges were purchased by Oasis. HIV p89.6 plasmid DNA was obtained from NIH AIDS reagent Program. Primers (HIV-1 forward primer: 5′-CAATGGCAGCAATTTCACCA-3′; HIV-1 reverse primer: 5′-GAATGCCAAATTCCTGCTTGA-3′) and HIV-1 probe (5′-[6-FAM] CCCACCAACAGGCGGCCTTAACTG [Tamra-Q]-3′) were purchased from IDT. Medidrop® Sucralose gel formulation was purchased from ClearH2O.

**Mouse blood and tissue processing.**

Peripheral blood samples were collected using retro-orbital sampling approximately 10-12 weeks post humanization, 4-6 weeks post HIV-1 infection, 4 weeks post ART and 12 weeks post potent ART (terminal phlebotomy). Red blood cells were lysed using Red Cell Lysis buffer solution (Biolegend) and remaining cells were washed with PBS. This was followed by blocking in FBS and further staining for flow cytometry analyses. Mice were fasted overnight prior to the day of sacrifice. They were administered an overdose of isoflurane anesthesia and perfused to remove blood prior to harvesting organs as described previously[1, 2]. Tissue samples were collected at necropsy and were either snap frozen in liquid nitrogen or were processed immediately for cell isolation and flow cytometry analysis. Tissues were weighted and <0.25 gr tissue samples were placed in 7 ml screwcap dissociation tubes with ceramic beads (Precellys) and were mechanically dissociated at power 3000 for one 20-second cycle using a Precellys 24 (Bertin technologies)[3]. The homogenate was filtered through a sterile 70 μm nylon filter (Fisher Scientific) and cells were then processed for flow cytometry. This dissociation achieved adequate homogenization of tissue with cell viability > 80% without the need for enzymatic digestion (that may reduce the yield of cells and affect several epitopes especially chemokine receptors[4]). For tissue lysates that were used for lipid or protein measurements, 100 mg of gut tissue samples were placed in 2 ml screwcap dissociation tubes with ceramic beads (Precellys) and were mechanically dissociated in methanol (lipids) or T-PER™ tissue protein extraction Reagent (Thermo Scientific) at power 5000 leaving samples to cool on ice between 1–2 repeated 20-second cycles as previously described[5].

**Sample preparation for determination of bioactive lipids.**

We prepared tissue, plasma, and lysate samples first by combination with methanol and the antioxidant butylated hydroxytoluene (BHT), and then by SPE using Oasis HLB cartridges. Post SPE, samples were reconstituted in methanol for LCMS analysis as previously described[6]. Briefly, lipids were extracted from 50-100 mg of intestinal tissue by first adding 500 μl MS-grade H_2_0 together with 50 μl of 20 ng/ml internal standard mix as well as a final concentration of 20 μM BHT. The tissue was homogenized, 1 ml of methanol was added, the tissue was vortexed, and the sample was spun down at 15000 RPM for 10 min at 4^0^C. The supernatant was removed and brought up to 10 ml with pH 3-4 HCl-acidified H_2_O. SPE was performed on an Oasis HLB 3 cc column in accord with the manufacturer’s protocol. The sample was eluted with 2 ml methanol, dried down under argon, and brought up again in 100 μl methanol for LCMS analysis. The final concentrations of internal standards in the samples were 10 ng/ml. Lipids were extracted from 100 μl of plasma by first adding 50 μl of 20 ng/ml internal standard mix together with 150 μl of methanol. BHT was added to a final concentration of 20 μM. The sample was vortexed and spun down at 15000 RPM for 10 min at 40C. The supernatant was combined with 1.8 ml of HCl-acidified H2O (pH = 3-4); SPE was performed on 3 cc Oasis HLB cartridges; and samples were eluted with methanol, dried down under argon, and brought up in 100 μl of methanol for LCMS analysis. Each sample preparation method was independently validated by determining precision and recovery, as described[6].

**MRM LC-MS/MS Pro-Inflammatory and Inflammation Resolving Lipid Signaling Panel**

We have previously developed a multiple reaction monitoring (MRM) method in negative ion mode coupled together with liquid chromatography that covers 39 distinct bioactive lipids, degradation products, and pathway markers[6]. We assigned each analyte to one of 19 distinct structurally identical or class-specific deuterated internal standards[6]. In the cases in which a particular analyte lacked a structurally identical internal standard, we assigned an internal standard that both shared the same basic structure and co-eluted within 0.5 min of the analyte in question. Final data was normalized to tissue weight, plasma volume, or for lysate protein concentration as determined by Bradford from a small aliquot of each lysate. We divided the bioactive lipids into pro-inflammatory, anti-inflammatory, and pro-resolving functional categories by literature search (see prior publication[6]), while further indexing these as being cyclooxygenase (COX) or LOX products and as being derived from the Arachidonic acid (AA), Docosahexaenoic acid (DHA) and Eicosapentaenoic acid (EPA)[6]. In the cases in which a particular analyte lacked a structurally identical internal standard, we assigned an internal standard that both shared the same basic structure and co-eluted within 0.5 min of the analyte in question. Chromatography was performed on an Agilent 1290 UHPLC system using a Phenomenex Kinetex C18 column (2.6 uM particle size, 2.1 mm ID x 150 mm) and gradient elution. Solvent A was 75/25/0.1 (H2O/acetonitrile/formic acid) and Solvent B was 100/0.1 (acetonitrile/formic acid), and the gradient was as follows: 0-8.5 min, 0-85%B; 8.5-9.5 min, 85-100%B; 9.5-10.5 min, 100%B; 10.5-12 min, 100-0% B; 12-14 min, 0%B; with an additional 1 min equilibration at the starting conditions prior to each run. The column oven was set to 40 deg C, while the flow rate was 350 ul/min. 5 ul of sample was injected for each run. Mass spectrographic analysis was performed on a SCIEX 5500 QTrap run in negative ion mode and controlled by Analyst 1.6.2 software. Two separate MRM transitions were determined for each compound, and DP, EP, CE, and CXP for each compound were all manually optimized by tuning on direct infusions of approximately 50 ng compound/ml 50%B[6]. The source settings were optimized by tuning on a mix of analytes while also flowing in 50%B at 350 ul/min, with temperature set at x, GS1 at x, GS2 at x, CAD gas a medium and CUR gas at 25. Scheduled MRM was employed, with the window for each MRM channel set at 60 seconds around the retention times (RT) of each compound[6]. LLOD, LLOQ, ULOQ, and linearity of response for each analyte were determined[6]. Data analysis was performed using MultiQuant software, with the concentration of each analyte being determined relative to its internal standard and against standard curves. Final data was normalized to tissue weight, plasma volume, media volume, or for lysate protein concentration as determined by Bradford from a small aliquot of each lysate.

**Flow cytometry**

For flow cytometry, single cell suspensions were incubated with antibodies that are described in the materials. For membrane antibody staining, intestinal cells were stained with surface markers. After 30 min, the cells were washed twice with fluorescence-activated cell sorting (FACS) buffer (PBS + 5% FBS). After a short spin, the cells were suspended in 200 μL of ice-cold PBS buffer and transferred to fresh tubes for FACS analysis. LSR Fortessa flow cytometer and FACSDiva software (BD Biosciences) were used for acquisition of flow cytometry data which were analyzed using FlowJo software. At least 1000 cells were acquired for each analysis, and each representative flow plot was repeated more than 3 times. Only live and singlet cells were chosen for analysis and gating (i.e., dead cells and aggregates were excluded).

**Statistics.**

Unless noted, error bars in all figures represent median and interquartile range (IQR). In the figures, p-values are presented for comparisons between treatment groups and controls and are denoted by asterisks. To pool mice from different cohorts, each measurement was first normalized to the uninfected controls of the cohort. Each analysis contains at least 4 biological replicates (number of mice). For flow analysis of cells, at least 100 events were acquired for the population of interest. For analysis of data that contains more than 2 groups, the Kruskal-Wallis test was performed to compare samples; if these comparisons had P value less than 0.05 then Mann-Whitney U tests were used to compare statistical difference between 2 groups. P values less than 0.05 by Kruskal-Wallis or Mann-Whitney were considered significant. For all correlations, Spearman's correlation coefficient was calculated. In the setting of exploratory approach we did not adjust for multiple comparisons since commonly-used multiple testing adjustment methods assume independence of tests, which in protein expression studies and in our explored pathways translates to a questionable assumption that all explored measures operate independently[7]. Instead consistency between 2 independent BLT models, direction, and magnitude of the correlation coefficient in conjunction with the nominal P values were considered in order to help distinguish true and false-positive findings. Consultation on statistical analysis was performed with the UCLA Biostatistics Department. All analyses were performed with Graphpad, version 7.0.

**REFERENCES SUPPLEMENTAL MATERIAL**

[1] Chattopadhyay A, Navab M, Hough G, Gao F, Meriwether D, Grijalva V, et al. A novel approach to oral apoA-I mimetic therapy. J Lipid Res. 2013;54:995-1010.

[2] Chattopadhyay A, Yang X, Mukherjee P, Sulaiman D, Fogelman HR, Grijalva V, et al. Treating the Intestine with Oral ApoA-I Mimetic Tg6F Reduces Tumor Burden in Mouse Models of Metastatic Lung Cancer. Sci Rep. 2018;8:9032.

[3] Welte T, Yu C, Zhang XH. Retrieval of Disseminated Tumor Cells Colonizing the Bone in Murine Breast Cancer Metastasis Models. J Mammary Gland Biol Neoplasia. 2015;20:103-8.

[4] Trapecar M, Khan S, Roan NR, Chen TH, Telwatte S, Deswal M, et al. An Optimized and Validated Method for Isolation and Characterization of Lymphocytes from HIV+ Human Gut Biopsies. AIDS Res Hum Retroviruses. 2017;33:S31-S9.

[5] Lofgren L, Forsberg GB, Stahlman M. The BUME method: a new rapid and simple chloroform-free method for total lipid extraction of animal tissue. Sci Rep. 2016;6:27688.

[6] Meriwether D, Sulaiman D, Volpe C, Dorfman A, Grijalva V, Dorreh N, et al. Apolipoprotein A-I mimetics mitigate intestinal inflammation in COX2-dependent inflammatory bowel disease model. J Clin Invest. 2019;130:3670-85.

[7] Stevens JR, Al Masud A, Suyundikov A. A comparison of multiple testing adjustment methods with block-correlation positively-dependent tests. PLoS One. 2017;12:e0176124.
